# A Case of Tubercular Pericardial Tamponade With Anti-tuberculosis Treatment-Induced Hepatitis

**DOI:** 10.7759/cureus.59050

**Published:** 2024-04-26

**Authors:** Daniel Hijam, Supongbenla Supongbenla, Doyen Soram

**Affiliations:** 1 General Practice, Regional Institue of Medical Sciences, Imphal, IND; 2 Internal Medicine, Regional Institute of Medical Sciences, Imphal, IND; 3 General Practice, Regional Institute of Medical Sciences, Imphal, IND

**Keywords:** att induced hepatitis, pericardiocentesis, cardiac tamponade, pericarditis, tuberculosis

## Abstract

Pericarditis can be a common complication of tuberculosis (TB) in developing countries like India. It is associated with fever, fatigue, and weight loss and can often be accompanied by shortness of breath and chest pain. Other common causes of pericardial effusion include malignancy, renal failure, autoimmune disease, and viral and bacterial infections. When the pericardial fluid is bloody, TB is likely to be present in developing countries. It can often get complicated with cardiac tamponade, which has a high mortality rate.

We present a case of a 55-year-old female with no co-morbidities who presented with shortness of breath, fatigue for two weeks, and chest pain for one week. She had no history of fever, chills, or rigour, and no history of TB contact. Clinical examination revealed low blood pressure with raised jugular venous pressure (JVP). Her electrocardiography (ECG) showed sinus tachycardia with a low-voltage complex. Echocardiography (ECHO) showed a large pericardial effusion, compromising ventricular function. We performed pericardiocentesis, drained 1.4 L of bloody fluid, and sent the pericardial fluid for analysis. Pericardial fluid adenosine deaminase (ADA) and cartridge-based nucleic acid amplification testing (CBNAAT) came positive for Mycobacterium TB. The patient was started on anti-tubercular treatment (ATT) and broad-spectrum antibiotics with drainage. Other routine investigations and autoimmune immune workups were normal. The patient also developed ATT-induced hepatitis, for which modified ATT was initiated. The patient improved clinically and symptomatically, was discharged, and was advised to follow up in the outpatient department (OPD).

## Introduction

Tuberculosis (TB) is one of the most common causes of pericarditis in developing countries [1]. In developed countries, it is rare in immunocompetent and human immunodeficiency virus (HIV)-negative individuals [2]. Patients can present with non-specific signs and symptoms like fatigue, fever, weight loss, cough, shortness of breath, and chest pain [3]. It can often present with cardiac tamponade if early diagnosis and treatment are not initiated. Cardiac tamponade is an accumulation of fluid in the pericardial space, leading to compromised cardiac activity and shock, which is fatal [4]. The three principal features of tamponade are hypotension, soft or absent heart sound, and raised jugular venous pressure (JVP). The most common causes of tamponade are idiopathic pericarditis and pericarditis secondary to neoplastic diseases, TB, or bleeding into the pericardial space after leakage from an aortic dissection, cardiac operation, trauma, or treatment with anticoagulants [5]. In this case report, a patient presented with cardiac tamponade due to tubercular pericarditis, compounded by anti-tubercular treatment (ATT)-induced hepatitis.

## Case presentation

**Figure 1:**
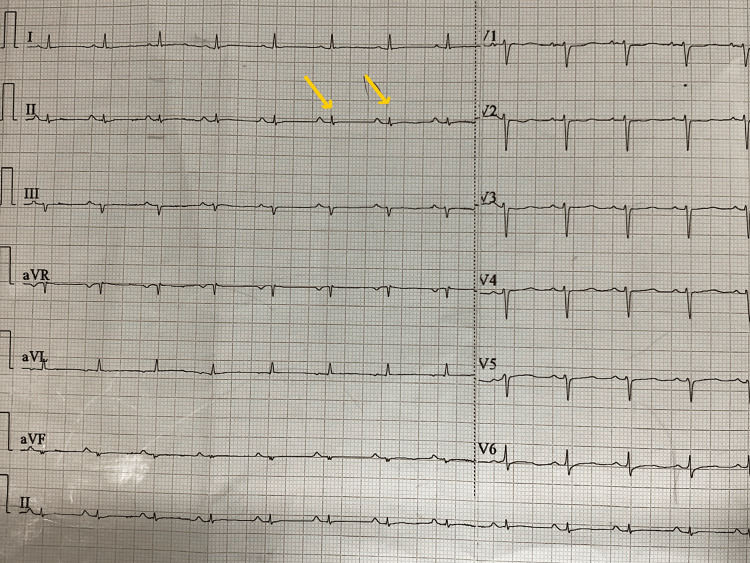
ECG showing sinus tachycardia with reduced QRS complex amplitude

**Figure 2:**
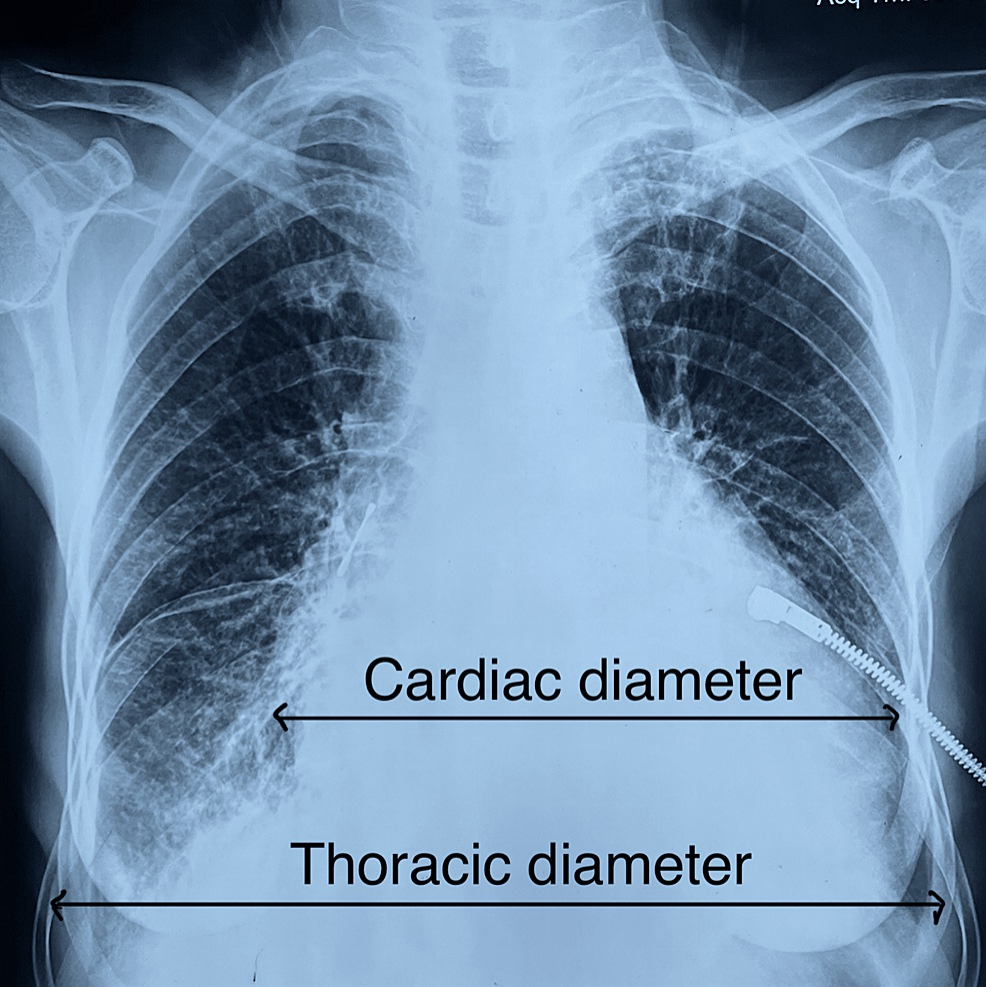
CXR showing increased cardiothoracic ratio suggestive of cardiomegaly CXR: chest X-ray

A 55-year-old female presented to the emergency department with a two-week history of fatigue, shortness of breath, and chest pain for one week. It was not associated with nausea, vomiting, coughing, or palpitations. She had no history of fever, chills, or rigours, and no history of TB contact. There was no history of weight loss or night sweats. She did not have any co-morbidities and no history of drug use for any illness. On assessment, she had a temperature of 37 °C, a blood pressure of −80/60 mm Hg, a pulse rate of 130 bpm, a SpO_2_ of 96% in room air, and a respiratory rate of 18/min. Clinical examination revealed raised JVP and soft heart sounds. Electrocardiography (ECG) showed sinus tachycardia with a reduced QRS complex amplitude (Figure 1), cardiac enzyme hs Trop I was within the normal limit, and chest X-ray (CXR) showed an enlarged cardiac silhouette (Figure 2).

**Figure 3:**
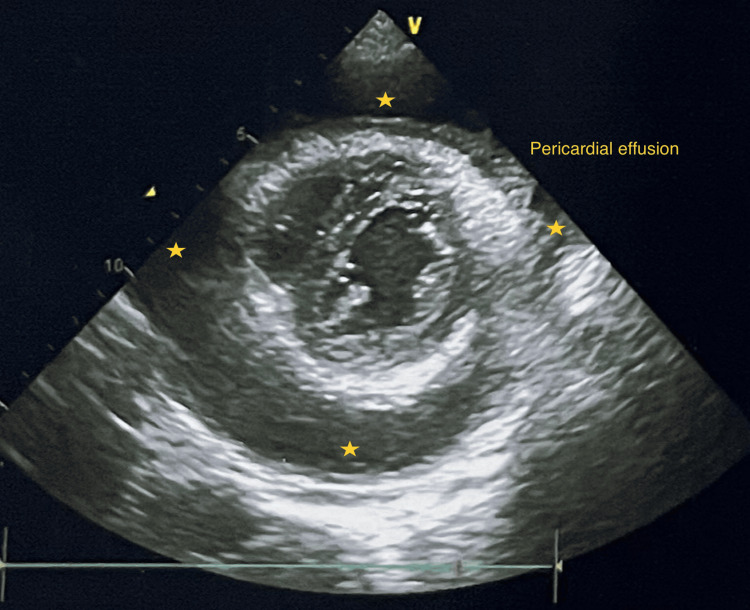
2D Echo showing pericardial effusion distributed all around the heart

**Figure 4:**
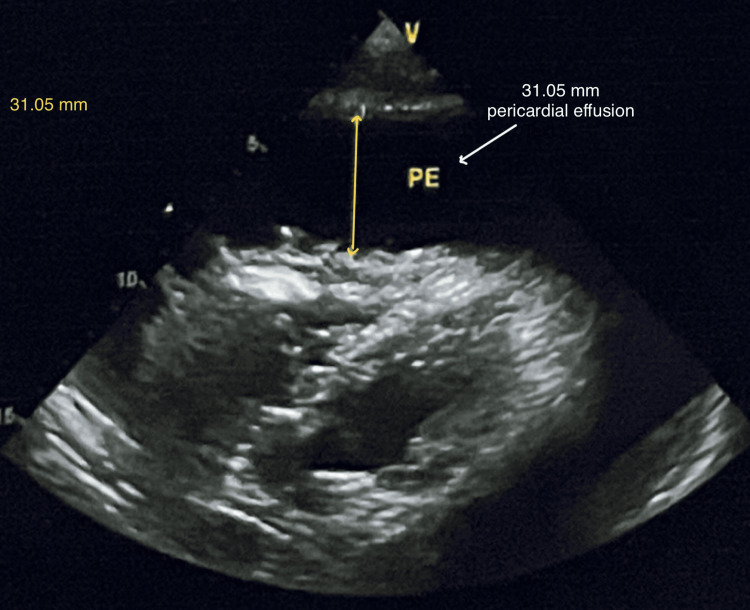
2D Echo showing massive pericardial effusion measured up to 31.05 mm

Other routine investigations were within the normal limit. 2D echocardiography (2D Echo) showed a large pericardial effusion, compromising ventricular function, suggesting cardiac tamponade with no features of infective endocarditis (Figures 3-4).

**Figure 5:**
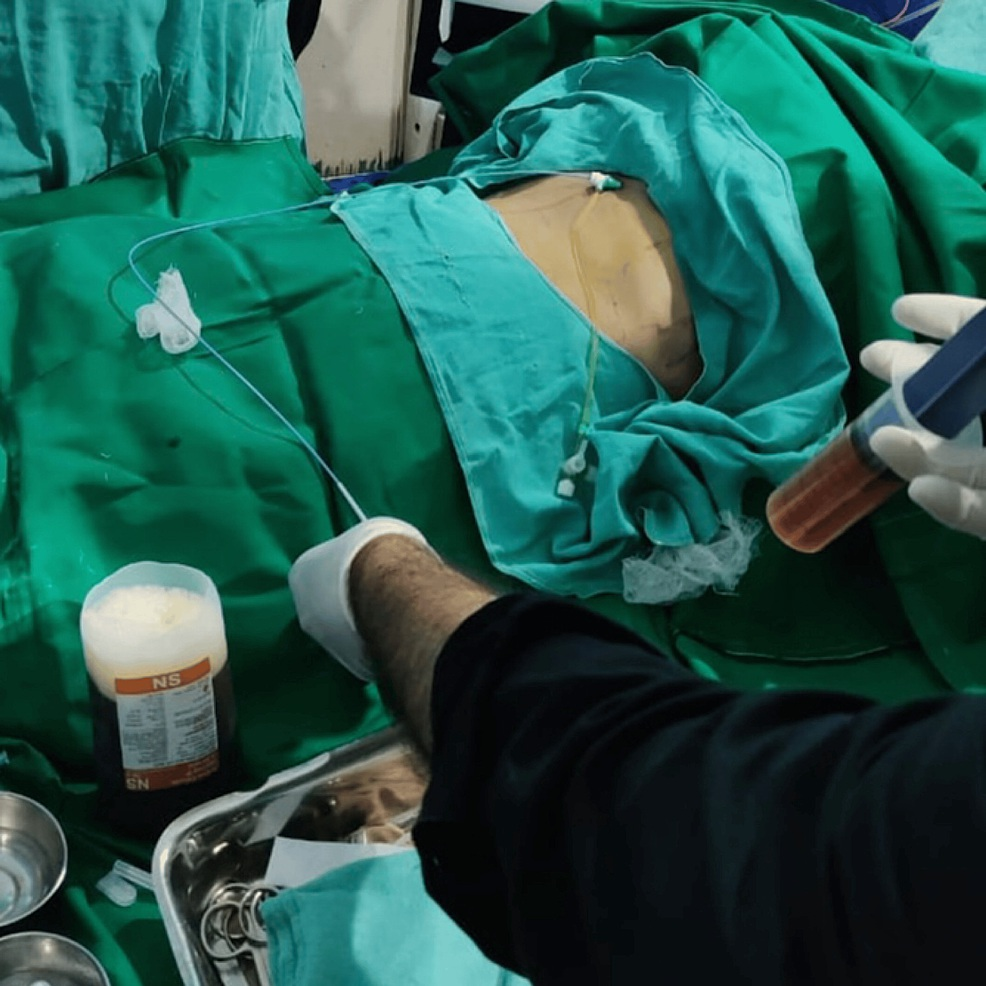
Pericardiocentesis showing pericardial bloody fluid

The patient was shifted to the intensive care unit (ICU), and emergency pericardiocentesis was done through a sub-xiphisternal approach under 2D Echo guidance. A 6F pigtail catheter was kept, bloody pericardial fluid of 1.4 L was drained, and a pericardial fluid analysis was sent (Figure 5).

Pericardial fluid analysis report showed adenosine deaminase (ADA) of 96.1 U/L (normal: <40 U/L), total cell count: 90 cells with 95% lymphocytes and 5% neutrophils, malignant cells: not seen, CBNAAT Mycobacterium TB positive with Rifampicin sensitivity indeterminate (Table 1).

**Table 1:** Pericardial fluid analysis on admission ADA: adenosine deaminase, CBNAAT: cartridge-based nucleic acid amplification testing, TB: tuberculosis

Pericardial fluid analysis	Value	Biological reference interval
Appearance	Haemorrhagic	Clear to pale yellow
Coagulum	Positive	Nil
Total protein (g/dL)	3.7	1.7–4.6
Glucose (mg/dL)	72	80–134
ADA	96.1 U/L	<40 U/L
Total cell count (10^6 ^cells/L)	90	35–2210
Lymphocytes (10^6 ^cells/L)	86	19–1634
Neutrophils (10^6 ^cells/L)	4	0–2
Malignant cell	Not seen	Nil
CBNAAT for Mycobacterium TB	Positive	Nil

The patient was started on ATT with broad-spectrum antibiotics and oral steroids, which included injection of piperacillin 4000 mg with tazobactam 500 mg intravenous thrice daily for seven days and oral prednisone 20 mg, one tablet once daily for seven days, respectively. We administered oral isoniazid (H) 300 mg, rifampicin (R) 450 mg, pyrazinamide (Z) 1500 mg, and ethambutol (E) 800 mg daily with pyridoxine supplementation. The patient developed severe nausea and vomiting by day 11 of treatment, and liver enzymes showed aspartate aminotransferase (AST) and alanine aminotransferase (ALT) more than three times the upper limit (AST>ALT). Jaundice was not visible clinically, and the prothrombin time-international normalized ratio (PT-INR) was high. In view of drug-induced hepatitis, ATT was therefore changed to modified ATT, i.e., injection streptomycin 750 mg, oral levofloxacin 750 mg, and ethambutol 800 mg per day. Hepatitis viral markers and HIV were negative. The patient was restarted with HRZE once the liver enzymes came down (Table 2 shows values of liver enzymes on different days of hospitalization). The pericardial drainage was carried out, yielding about 120-100 ml for the initial seven days and gradually decreasing to less than 10 ml per day, which was removed after three weeks. The blood, pleural fluid, and urine cultures were sterile. The USG abdomen showed normal findings; autoimmune, thyroid, and malignancy workups were negative. The patient improved clinically and symptomatically, got discharged, and was advised to follow up in the OPD.

**Table 2:** Values of liver function test on different days of hospitalization LFT: liver function test; S. T. Bil: serum total bilirubin, S. T. Prot.: serum total protein, S. albumin: serum albumin; S. globulin: serum globulin; S. AST: serum aspartate transaminase; S. ALT: serum alanine transaminase; S. ALP: serum alkaline phosphatase; S. GGT: serum gamma-glutamyl transpeptidase

LFT	Before ATT	Day 11 of ATT	After 1 month of modified ATT	Reference intervals
S. T. bil	0.3	0.8	0.6	0.2–1.2 mg%
S. D. bil	0.1	0.2	0.2	<0.2 mg%
S. T. prot	5.9	6.3	6.6	6–8 g%
S. albumin	3.4	2.4	3.5	3.5–5.5 g%
S. globulin	2.5	3.9	3.1	1.8–3.6 g%
S. AST	40	919	27	<40 IU
S. ALT	35	324	23	<40 IU
S. ALP	178	159	225	30–120 IU
S. GGT (female)	61	83	211	7–32 IU

## Discussion

Mycobacterium TB presenting with pericardial disease complicated by cardiac tamponade is rare in developed countries, although it occurs more frequently in the context of immunosuppression [2]. In developing countries, it is still prevalent and can be life-threatening, even for immunocompetent patients [6]. Patients with tuberculous pericarditis are often associated with fever, fatigue, anorexia, weight loss, and night sweats, and on the extreme end of the spectrum, it can be associated with cardiac tamponade with a high mortality risk. It is most often caused by direct lymphatic spread or haematogenous seedlings. Risk factors include immunocompromised, elderly, and underlying co-morbid conditions. Our patient has no previous history of TB or TB contact, no underlying co-morbidities, and no history of trauma. In haemodynamically unstable patients, an emergency 2D-Echo should be done to rule out cardiac tamponade [7].

Diagnosis is made by pericardial fluid studies, including polymerase chain reaction (PCR), culture, and pericardial biopsy [8]. In our patient, pericardial fluid was bloody with high ADA activity (>40 U/L), and CBNAAT for Mycobacterium TB was positive. Management of tuberculous pericarditis consists of two months of Isoniazid, Rifampicin, Pyrazinamide, and Ethambutol, followed by four months of Isoniazid and Rifampicin (a total of six months of treatment) [9]. In our patient, oral Isoniazid 300 mg, Rifampicin 450 mg, Pyrazinamide 1500 mg, and Ethambutol 800 mg per day were given with pyridoxine supplementation and pericardial drainage.

Hepatotoxicity is one of the most common adverse drug reactions (ADR). Other ADRs include gastrointestinal and neurological disorders [10]. The incidence of drug-induced liver injury in India is said to be between 8% and 36% [11]. Our patient developed drug-induced hepatitis on day 11 of ATT and therefore changed to modified ATT: injection of streptomycin 750 mg, oral levofloxacin 750 mg, and ethambutol 800 mg. The patient was restarted with HRZE once liver enzymes were normalised. In this case, a patient with no co-morbidities came with tubercular pericardial effusion complicated by cardiac tamponade, requiring emergency pericardiocentesis and daily pericardial drainage. The patient was started on ATT, following which ATT-induced hepatitis developed, requiring supportive management, intensive care, and monitoring.

## Conclusions

In patients presenting with pericardial effusion, tuberculosis has to be tested in developing countries like India, and if complicated with cardiac tamponade, emergency pericardiocentesis has to be done. Optimal management requires a combination of a drug-sensitive ATT regimen and pericardial drainage. Liver function tests along with other routine tests should be carried out before initiating ATT, and monitoring has to be done during the treatment as hepatotoxicity is one of the most common ADRs. Early diagnosis and treatment significantly improve the outcome.
